# Evidence of Expanded Host Range and Mammalian-Associated Genetic Changes in a Duck H9N2 Influenza Virus Following Adaptation in Quail and Chickens

**DOI:** 10.1371/journal.pone.0003170

**Published:** 2008-09-09

**Authors:** Md Jaber Hossain, Danielle Hickman, Daniel R. Perez

**Affiliations:** Department of Veterinary Medicine, University of Maryland, College Park and Virginia-Maryland Regional College of Veterinary Medicine, College Park, Maryland, United States of America; U.S. Naval Medical Research Center Detachment/Centers for Disease Control, United States of America

## Abstract

H9N2 avian influenza viruses continue to circulate worldwide; in Asia, H9N2 viruses have caused disease outbreaks and established lineages in land-based poultry. Some H9N2 strains are considered potentially pandemic because they have infected humans causing mild respiratory disease. In addition, some of these H9N2 strains replicate efficiently in mice without prior adaptation suggesting that H9N2 strains are expanding their host range. In order to understand the molecular basis of the interspecies transmission of H9N2 viruses, we adapted in the laboratory a wildtype duck H9N2 virus, influenza A/duck/Hong Kong/702/79 (WT702) virus, in quail and chickens through serial lung passages. We carried out comparative analysis of the replication and transmission in quail and chickens of WT702 and the viruses obtained after 23 serial passages in quail (QA23) followed by 10 serial passages in chickens (QA23CkA10). Although the WT702 virus can replicate and transmit in quail, it replicates poorly and does not transmit in chickens. In contrast, the QA23CkA10 virus was very efficient at replicating and transmitting in quail and chickens. Nucleotide sequence analysis of the QA23 and QA23CkA10 viruses compared to the WT702 virus indicated several nucleotide substitutions resulting in amino acid changes within the surface and internal proteins. In addition, a 21-amino acid deletion was found in the stalk of the NA protein of the QA23 virus and was maintained without further modification in the QA23CkA10 adapted virus. More importantly, both the QA23 and the QA23CkA10 viruses, unlike the WT702 virus, were able to readily infect mice, produce a large-plaque phenotype, showed faster replication kinetics in tissue culture, and resulted in the quick selection of the K627 amino acid mammalian-associated signature in PB2. These results are in agreement with the notion that adaptation of H9 viruses to land-based birds can lead to strains with expanded host range.

## Introduction

The first isolation of a H9N2 subtype avian influenza virus was in 1966 in the U.S. [1]. Since then in North America, H9N2 avian influenza viruses have been found mainly in shorebirds and wild ducks, with no evidence of permanent lineages of these viruses in land-based poultry. In contrast, H9N2 viruses are endemic in Asia and have been isolated from domestic poultry in many countries [2]–[7]. More importantly, H9N2 viruses have occasionally transmitted from land-based poultry to mammals, including humans. Mild respiratory disease in humans was reported in 1999 in Hong Kong and mainland China and again in 2003 in Hong Kong [8]–[10]. In addition, infection of pigs with H9N2 viruses occurred on at least two occasions in 1998 and 2004 [9], [11], [12]. Interestingly, some of the currently circulating H9N2 strains have acquired human-like receptor specificity, i.e. they bind efficiently to sugar moieties terminated with α2,6 sialic acids. We have recently shown that Leucine (Leu) at position 226 in the receptor-binding site (RBS) of H9N2 viruses allows them to display a human influenza-like virus phenotype in human airway epithelial cells (HAE) [13]. H9N2 viruses with Leu-226 infect mostly nonciliated cells in HAE cultures and can spread from cell to cell forming foci of infection in a manner similar to human influenza viruses [13]. In contrast, H9N2 viruses with glutamine-226 in the RBS are more restricted for growth in HAE cells, suggesting that a single amino acid change can have profound implications for the host range of these viruses. Further evidence for the expansion of the host range of contemporary Asian H9N2 viruses is the observation that these strains can replicate efficiently in mice without prior adaptation [14]. Lethality for mice was observed for some H9N2 strains isolated from land-based birds, including chickens and quail [14], [15]. In contrast, early H9N2 field strains isolated from chickens replicate poorly or not at all in mice [16]. Furthermore, we have recently shown that Leu226-containing H9N2 viruses can replicate in ferrets and transmit to direct contacts [17].

To date, the mechanism of adaptation and interspecies transmission of H9N2 viruses is poorly understood. Accumulating data suggests that among the land-based poultry, quail play a crucial role for the genesis of influenza viruses. We have previously shown that quail provide an environment where most influenza subtypes are able to replicate, primarily in the respiratory tract [18], [19]. We have also observed that quail are more susceptible than chickens to infection with the duck H9N2 viruses isolated in China in the late 1970's [20] . Subsequently, we discovered that in quail the receptors for both avian (SAα2,3) and human (SAα2,6) influenza viruses are abundantly distributed [21]. These findings led us to hypothesize that quail could play an important role as intermediate hosts that permit adaptation of influenza viruses from wild aquatic birds and the generation of variant viruses that can cross to other species, such as chicken, pigs, or humans. In addition, quail could provide an environment in which avian-mammalian reassortant viruses could be amplified, thereby increasing the likelihood of interspecies transmission [22]. In fact, phylogenetic analyses of a broader sample of avian influenza viruses isolated from live-poultry markets in Hong Kong in 1997 showed that the internal genes of the human and avian influenza H5N1 isolates were most closely related to a single H9N2 quail influenza A virus isolated in the area [23].

In order to understand the molecular basis of adaptation and transmission of H9N2 viruses in different animal species in nature, we developed a laboratory-based model to adapt an H9N2 virus to land-based poultry. Our results suggest that laboratory adaptation of a duck H9N2 virus through serial lung passages in quail and chickens generated a variant virus that is capable of efficiently replicating and transmitting in chickens. More importantly, the adapted virus gained the ability to replicate efficiently in mice when inoculated intranasally. The results from this laboratory-based adaptation study mimic the ongoing activities of H9N2 viruses in nature and highlight the potential role of land-based poultry in the genesis of influenza viruses with expanded host range.

## Results

### Replication and transmission of a duck H9N2 virus in quail and chickens

Initial studies in our laboratory confirmed previous findings and showed that the WT702 virus replicates and transmits in Japanese quail but replicates poorly and does not transmit in White leghorn chickens [20]. Our results indicated that infected quail shed virus from 1 to 9 days post-infection (dpi) (Table 1). The WT702 virus transmitted efficiently to direct contact quail in which 3 out 3 were positive for virus isolation starting at day 3 post-infection. In contrast, duration of virus shedding in chickens infected with the WT702 virus was not observed beyond 5 dpi with only the inoculated chickens shedding virus through the respiratory tract (Table 2). None of the contact chickens shed any virus and no evidence of infection was observed by HI assays (Table 2 and data not shown).

**Table 1:** Replication and transmission of H9N2 viruses in Japanese quail.

Groups	Days post-infection*					
	1	3	5	7	9	11
Infected	3/3	3/3	3/3	3/3	2/3	0/3
Contact	NA	3/3	3/3	2/3	1/3	0/3

* , Data from tracheal swabs.

NA, Not applicable.

**Table 2:** Replication and transmission of H9N2 viruses in white leghorn chickens.

Virus/Group	Days post-infection													
	1		3		5		7		9		11		13	
	T	C	T	C	T	C	T	C	T	C	T	C	T	C
**WT702** *														
Infected	6/6	0/6	6/6	0/6	4/6	0/6	0/6	0/6	0/6	0/6	NT	NT	NT	NT
Contacts	NA	NA	0/6	0/6	0/6	0/6	0/6	0/6	0/6	0/6	NT	NT	NT	NT
**QA23** †														
Infected	12/12	0/12	12/12	0/12	11/12	5/12	4/12	4/12	0/12	3/12	NT	3/12	NT	0/12
Contacts	NA	NA	0/12	0/12	2/12	0/12	3/12	0/12	4/12	0/12	2/12	NT	0/12	NT
**QA23CkA10** *														
Infected	6/6	0/6	6/6	1/6	6/6	5/6	2/6	6/6	1/6	2/6	0/6	1/6	NT	0/6
Contacts	NA	NA	2/6	0/6	5/6	0/6	6/6	0/6	5/6	1/6	2/6	1/6	0/6	0/6

* , Data from two independent studies.

† , Data from four independent studies; NT, Not tested; NA, Not applicable; T and C indicates tracheal and cloacal swabs, respectively.

### Laboratory adaptation in quail of the WT702 virus leads to a virus that replicates and transmits readily in quail and replicates more efficiently in chickens

In order to make the WT702 virus more transmissible in chickens we followed a strategy that we have previously employed for adaptation of a mallard H2N2 virus in quail and chickens [24]. We must note that despite 6 independent attempts using groups of 3 chickens per study, we failed to consistently establish an infection and detect any virus in the lung homogenates of chickens infected with the WT702 virus at 3 dpi. In contrast, the WT702 was readily detected in the lung homogenates of quail infected under similar conditions, and the lung homogenates were used to obtain a virus better adapted to quail. We speculated that the quail-adapted virus could potentially be better adapted for replication in chickens. After 23 passages in quail, we obtained a virus (QA23) that was more efficient at replicating and transmitting in chickens than the WT702 virus or any of the previous quail-passaged viruses (Table 2). Out of 12 chickens infected with QA23, virus shedding through the trachea was observed in 11 of these chickens and with 5 chickens virus was detected in the cloaca at 5 dpi. Transmission studies indicated that the QA23 virus was partially transmissible in chickens. Out of 12 direct contact chickens, 4 chickens showed tracheal virus shedding at day 9, And two contact chickens were positive until day 11. The contact chickens were negative for virus shedding through the cloaca. Our studies indicate that serial lung passages of the WT702 virus in quail improved the virus's ability to replicate in chickens, where it was partially transmissible. Because of the partial transmission of the QA23 virus in chickens, we further adapted the QA23 virus through serial lung passages in chickens. After 10 serial chicken lung passages of the QA23 virus, we prepared a stock of the virus by inoculating the 10th passaged chicken lung homogenate into embryonated chicken eggs; the virus was designated QA23CkA10 (Table 2). Efficient replication and transmission in chickens of the QA23CkA10 virus was observed. All 6 chickens infected with the QA23CkA10 virus were positive for tracheal virus shedding by 5 dpi. Two of the infected chickens shed virus until day 7 and 1 chicken shed virus until day 9. In addition, the infected chickens started shedding virus through the cloaca as early as 3 dpi and 1 chicken continued to shed virus until day 11. Transmission of the virus to direct contact chickens started as early as 3 dpi and all direct contact chickens were positive for tracheal virus shedding by 7 dpi. Two of the direct contact chickens continued to shed virus until day 11. One contact chicken shed virus through the cloaca at 9 and 11 dpi. HI antibody titers in the sera of the direct contact chickens at 2 weeks post-contact confirmed the efficient transmission of the QA23CkA10 (not shown). These results indicate that serial lung passages of the QA23 virus in chickens produced a virus with improved fitness for replication and transmission in chickens. It must be noted that we did not establish the mode of transmission of these viruses, as birds were in the same cage sharing the same food and drinking water. Nevertheless, these studies suggest that our approach recapitulates the early natural events that led to the emergence of H9N2 viruses in land-based birds.

### Laboratory adaptation leads to H9N2 viruses with improved replication in the lungs of quail and chickens

**Figure 1:**
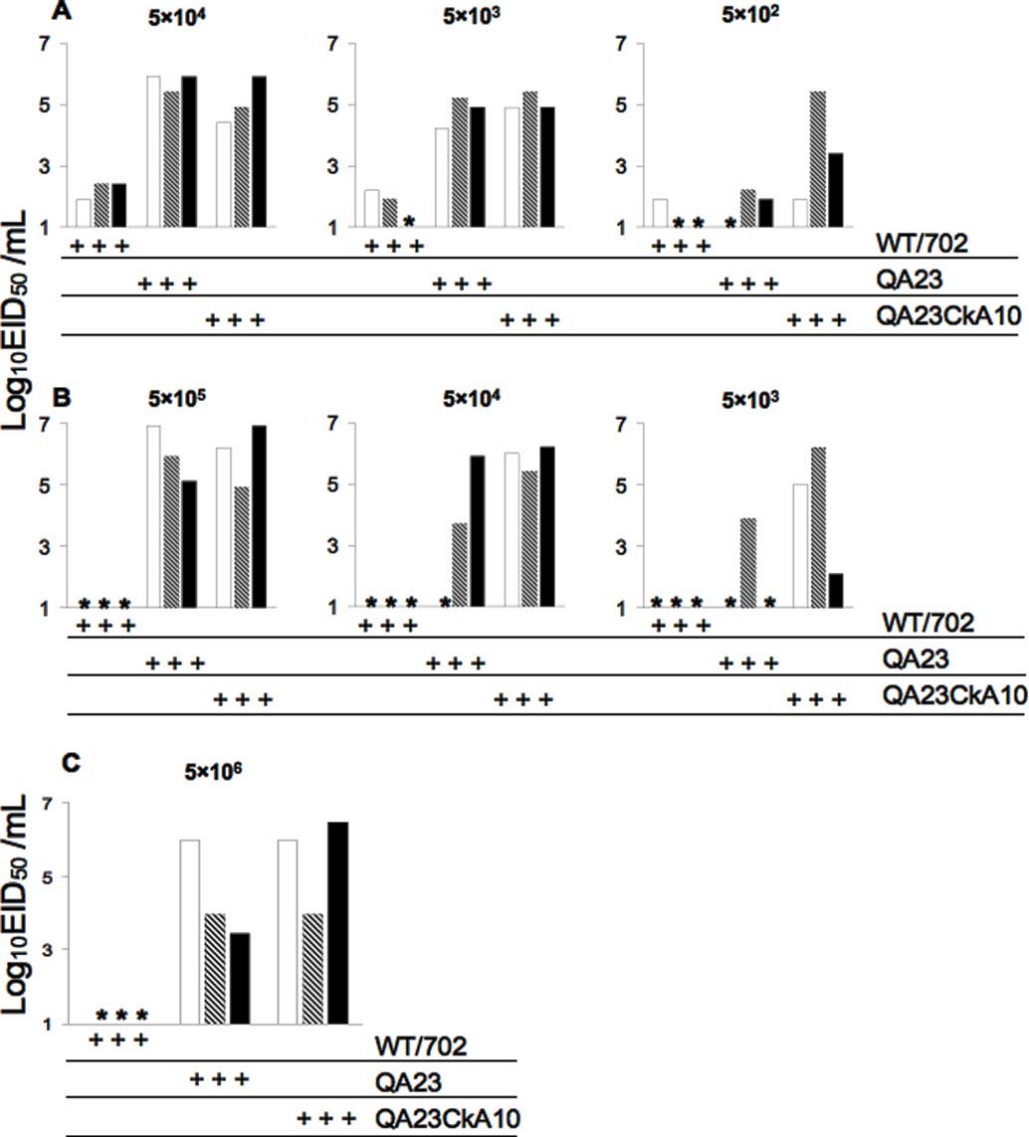
Replication of H9N2 duck, quail- or chicken-adapted viruses. A) Quail and B) chickens were infected with the indicated doses (in EID_50_) of WT702, QA23, or QA23CkA10 viruses (set of birds infected with given virus are marked as +++). Each bar corresponds to virus titers in lung homogenates of individual birds collected at 3 dpi. C) Chickens infected with the indicated dose of WT702, QA23, or QA23CkA10 viruses (set of birds infected with given virus are marked as +++). At 3 dpi, chickens were sacrificed and intestine collected to determine virus titers. * indicates no virus detected in the undiluted samples. Virus titers presented as log_10_EID_50_/mL.

In order to better characterize the replication efficiency of the adapted H9N2 viruses in quail and chickens, we performed infectious dose 50 (ID_50_) assays and analyzed tracheal and lung virus titers with the three viruses, WT702, QA23, and QA23CkA10. As shown in Table 3, the quail infectious dose (QID_50_) for the WT702, QA23 and QA23CkA10 viruses were similar, between 1×10^2^ and 5×10^1^ EID_50_. In contrast, the chicken infectious dose 50 (CID_50_) required 1,000-fold more WT702 virus (5×10^5^ EID_50_) than for the QA23 and QA23CkA10 viruses (5×10^2^ and 2×10^2^ EID_50_, respectively). We then analyzed the virus titers in the lungs of quail and chickens infected with different doses of the WT702, QA23 and QA23CkA10 viruses (Fig. 1). Lungs were collected at 3 dpi, and virus titers were measured in lung homogenates. The results of these studies indicate that the QA23- and QA23CkA10-adapted viruses replicate more efficiently in the lungs of quail (Fig. 1A) and chickens (Fig. 1B) than the WT702 virus. These studies also indicate that the QA23CkA10 virus replicates better in the lungs of quail and chickens than the QA23 virus. Overall, these studies suggest that laboratory adaptation of a duck H9N2 virus through serial lung passages in quail and chickens has resulted in strains more efficient at causing respiratory infections and transmitting in the latter two species.

**Table 3:** Quail and Chicken infectious doses of the adapted viruses.

Viruses	Infectious dose (ID50)*	
	Quail (QID50)	Chicken (CID50)
WT702	1×10^2^	5×10^5^
QA23	1×10^2^	5×10^2^
QA23CkA10	5×10^1^	2×10^2^

* , Infectious doses were determined from the tracheal swab taken at 3 dpi and expressed in EID50.

### The chicken H9N2-adapted virus has improved replication in the chicken's intestinal tract

It is commonly accepted that avian influenza viruses replicate predominantly in the intestinal tract of waterfowl and are secreted in large amounts through feces. Transmission of the virus is thus perpetuated through a fecal-oral route. In land-based birds, transmission of avian influenza viruses may include a combination of aerosol and fecal-oral routes. In order for transmission to occur through the fecal-oral route in land-based birds, the virus has to be able to replicate in the intestinal tract of the bird. To determine the extent of intestinal virus replication of the duck H9N2 virus after laboratory adaptation in chickens, chickens were infected with 5×10^6^ EID_50_ of the WT702, QA23 or QA23CkA10 viruses; 3 days later intestinal samples were taken and virus titers were determined. The quail- or chicken-adapted viruses were readily detected in the intestinal tract of chickens (Fig. 1C). In contrast, we could not detect any virus in the intestine of chickens inoculated with the WT702 virus. These studies indicate that the quail- and chicken-adapted viruses not only replicate better than WT702 in the respiratory tract of quail and chickens, but also replicate better than WT702 in the intestine of chickens. Thus, our laboratory adaptation strategy has also resulted in the expansion of the tissue tropism of the duck H9N2 virus in chickens.

### Molecular changes in the genome of quail- and chicken-adapted H9N2 viruses

To characterize the molecular features that allowed the duck H9N2 virus to become adapted to quail and chickens, we compared deduced amino acid sequences from the complete open reading frames of the WT702, QA23 and QA23CkA10 viruses (Tables 4 and 5). Details of the number of synonymous and nonsynonymous nucleotide changes are presented in Table 6. Comparative sequence analysis revealed 14 amino acid substitutions throughout the genome and one deletion in the NA stalk region in the QA23 virus when compared to the WT702 virus. Similar analysis showed 17 amino acid substitutions throughout the genome and one deletion in the NA stalk region in the QA23CkA10 virus when compared to the WT702 virus. The HA gene of the QA23 virus differed from the WT702 virus in the HA1 region at position 29 (T29A, 40 in the H3 HA) opposite to the peptide fusion domain, in the HA2 region at positions 43 (E43K, position 43 in the H3 HA numbering system) within the peptide fusion region, and 133 (D133N, 133 in the H3 HA) at the base of the molecule in close proximity to the viral membrane (Table 4). The HA gene of the QA23CkA10 virus carries the same three amino acid mutations found in the QA23 virus and an additional two amino acids substitutions in the HA1 region at positions 226 (V226I, 236 in the H3 HA) at the base of the globular domain and 319 (N319T, 327 in the H3 HA) within HA1/HA2 cleavage recognition sequence. All potential glycosylation sites remained unchanged in the HA of the adapted viruses. Two of those positions are located in regions previously recognized as potentially involved in the cross-species transmission of H9N2 viruses in land-based birds and possibly mammals [20]. The QA23 and QA23CkA10 share the same 21-amino acid deletion in the stalk region of the NA molecule (positions 58–78), suggesting that such deletion arose during adaptation of WT702 in quail and was maintained during the adaptation of the virus in chickens (Table 4). In addition to the deletion, a single amino acid change at position 79 (P79S, numbering based on the WT702 virus) was also observed in the NA of the adapted viruses. The QA23 virus was not cloned prior to adaptation in chickens and thus the possibility existed that a virus with a complete NA gene could have been present and selected out during the process of adaptation in chickens; however, this was not the case.

**Table 4:** Comparison of amino acid changes in the surface proteins of WT702, QA23 and QA23CkA10 1 viruses with other influenza viruses from different animal species.

Gene	AA position	Corresponding AA position in H3 HA	H9N2					
			WT702	QA23	QA23CkA10	Avian*	Human†	Swine†
HA								
HA1	29	40	T	A	A	A,T	A	A
	226	236	V	V	I	V,I,L,G	V	V
	319	327	N	N	T	S,K,G,D,N,A,Y,V	N	N
HA2	43	43	E	K	K	K,E,R	K	K
	133	133	D	N	N	D,S,N	D	D
NA	58–78	-	IERNVTEIVYLNNTTIERKIC	Deleted	Deleted	-	-	-
	79	-	P	S	S	P,S,L	P	P
M2	39	-	I	T	T	I,M,T	I,T	T,I

* , Data are from the alignment of 214 to 400 sequences available in the influenza sequence database.

† , Data are from the alignment of all the sequences available in the influenza sequence database.

- , Not applicable.

**Table 5:** Comparison of amino acid changes in the internal proteins of WT702, QA23, QA23CkA10 viruses with other influenza viruses from other animal species.

Proteins	Position	H9N2						Avian* (Any subtype)	Human H5N1†	Human (Others)*
		WT702	QA23	QA23CkA10	QA23ML-1	Human†	Swine†			
PB2	214	K	K	R	K	K	K	K, R, E	K	K, R, E
	470	S	C	S	C	S	S	S, N	S	S, N
	627	E	E	E	***K***	E	E	E, k	E, K	K
	758	I	T	I	T	I	I	I, T	I	I, T
PB1	703	S	S	C	S	S	S	S	S	S
PA	549	L	I	I	I	L	L	L, I, F	L	L, I
NP	109	I	I	T	I	I	I	I, V, T	I	V, I
	232	T	T	I	T	T	T	T	T	T,A
M1	168	T	I	I	I	T, I	T	T, I	I, T	T, I
NS1	106	M	T	T	T	I, M	I, M	M, I	M, I	M, I
	217	K	E	E	E	K	K, E, L	K, N, R	N, K, D	K, T, N, E
	219	K	E	E	E	K	K	K, E	K	K, E
NEP	100	M	I	I	I	M	M	M, L, V, I	M	M, I

* , Data are from the alignment of 500 sequences available in the influenza sequence database.

† , Data are from the alignment of all the sequences available in the influenza sequence database.

**Table 6:** Comparison of nucleotide changes among the genomes of WT702, QA23 and QA23CkA10 viruses.

Genes	Position	WT702	QA23	QA23CkA10	QA23ML-1
PB2	444*	T	T	G	T
	668	A	A	G	A
	828*	T	C	T	C
	873*	G	G	A	G
	1435	A	T	A	T
	1906	G	G	G	A
	2292*	G	G	A	G
	2300	T	C	T	C
PB1	669*	G	A	A	A
	2131	A	A	T	A
PA	426*	A	A	G	A
	969*	C	T	C	T
	1669	C	C	A	C
	1779*	T	T	C	T
	2061*	A	A	G	A
HA	19†	C	T	T	T
	172	A	G	G	G
	763	G	G	A	G
	1043	A	A	C	A
	1174	G	A	A	A
	1230*	G	G	G	A
	1444	G	A	A	A
	1725†	C	A	A	A
NP	371	T	T	C	T
	564*	G	A	A	G
	740	C	C	T	C
NA	192–254	COMPLETE	DELETION	DELETION	DELETION
	255	C	G	G	G
	808*	T	T	C	T
M	412*	T	C	T	T
	499*	G	G	G	A
	528	C	T	T	T
	625*	G	G	G	T
	829	T	C	C	C
NS	343	T	C	C	C
	675	A	G	G	G
	681	A	G	G	G
	778	G	A	A	A

* , Indicates synonymous mutations

† , indicates nucleotide changes in non-coding region

With respect to the internal components of the virus, amino acid mutations were identified in the structural and nonstructural proteins (Table 5). The only exception was in the region encoding PB1F2, which showed no amino acid substitutions. Nine amino acid mutations arose during serial passages of a duck H9N2 virus in quail (QA23) and 6 after adaptation of the QA23 virus in chickens (QA23CkA10). The PB2 protein of the QA23 virus showed two amino acid differences compared to the WT702 virus at positions 470 (S470C) and 758 (I758T). Interestingly, after adaptation to chickens, the QA23CkA10 virus reverted back to the wild type sequences at these two locations (470 and 758) in PB2, although it acquired a new amino acid substitution at position 214 (K214R) compared to either the QA23 or the WT702 viruses. A single amino acid substitution was observed in the PB1 protein of the chicken-adapted QA23CkA10 virus at position 703 (S703C). The serine to cysteine mutations observed in the PB2 (S470C) of the QA23 virus and in the PB1 (S703C) of the QA23CkA10 virus may be unique to these adapted virus, since there are no influenza sequences in GenBank with similar mutations. However, these mutations are not artifacts since both the QA23 and QA23CkA10 viruses were plaque purified; three independent plaques were processed for RT-PCR and sequencing, which resulted in the identical mutations. These results suggest genomic instability or flexibility among influenza viruses, which may contribute to their successful adaptation to other animal species. A single amino acid substitution was noticed at position 549 (L549I) in the PA protein of the QA23 and QA23CkA10 viruses. There was also a single amino acid substitution observed in the M1 (T168I), M2 (I39T), and NEP (M99I) proteins of the QA23 virus, which were maintained in the QA23CkA10 virus. While no amino acid mutations were observed in the NP of the QA23 virus, two substitutions were observed in the NP of the QA23CkA10 virus at positions 109 (I109T) and 232 (T232I). The NS1 protein was the internal viral product with the most number of amino acid substitutions; 3 mutations were identified at positions 106 (M106T), 217 (K217E), and 219 (K219E) in the QA23 virus and were maintained in the QA23CkA10 virus. The mutations in the NS1 protein fall within regions of previously predicted functions: The M106T mutation is likely to modulate binding to the cellular cleavage and polyadenylation specificity factor (CPSF) [25] whereas the K217E and K219E mutations are within the nuclear targeting of the molecule [26]. All other mutations identified throughout the genome are located in regions with unknown activity and/or interaction function. However, except for the two cysteine residues found in PB2 and PB1, the other mutations have been previously identified in at least one other strain of the more than 500 avian influenza and 500 human influenza sequences deposited in GenBank that were utilized in our analysis (Tables 4, 5 and 6). These results suggest that our strategy of laboratory adaptation of influenza viruses in different bird species mimics mutations found in viruses that have emerged naturally.

### Adaptation of a duck H9N2 virus in quail and chickens leads to a virus better adapted to replicate in mice: Rapid selection in vivo of the E627K mutation in PB2 of the progeny virus

**Figure 2:**
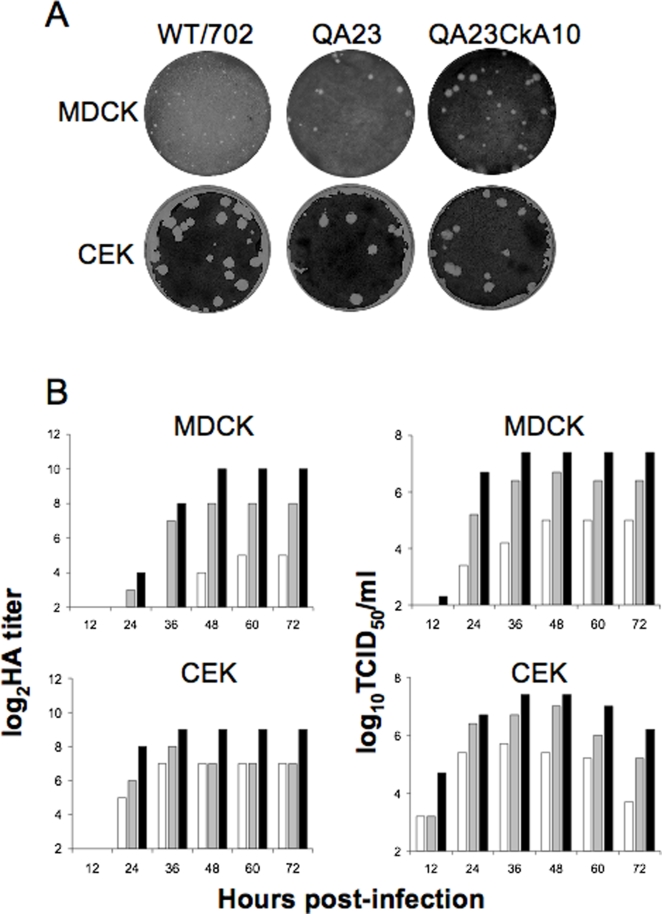
Plaque morphology and growth kinetics of H9N2 viruses in MDCK and CEK cells. A) Confluent monolayers of MDCK or CEK cells were infected with the corresponding viruses and maintained at 37°C in agar-maintenance medium supplemented with TPCK-trypsin. Cells were stained with crystal violet at 4 (MDCK) and 2 (CEK) dpi. B) Growth kinetics in confluent monolayer of MDCK (top panels) or CEK (bottom panels) infected with WT702 (open bar), QA23 (shaded bar) or QA23CkA10 (black bar) viruses at an input m.o.i of 0.001. Infected cells were maintained at 37°C in maintenance medium supplemented with TPCK-trypsin. Culture supernatants were collected at different hpi and virus titers measured by HA assay (left panels) or TCID_50_ in CEK cells (right panels).

H9N2 outbreaks in poultry in Asia have led to the emergence of strains with the capacity to cause mild respiratory disease in humans and that are able to replicate in pigs and mice without prior adaptation. The complexities associated with the emergence of such strains make it difficult to establish which avian species are capable of providing an environment, which allows these viruses to expand their host range. Thus, we were interested in determining whether the adaptation of a duck H9N2 virus in quail and chickens would result in strains better adapted to replicate in mammals, particularly mice. Female BALB/c mice were inoculated intranasally with different doses of the WT702, QA23 or QA23CkA10 viruses. Mice were monitored for the next 4 days for signs of disease, changes in body weight and overall wellbeing. Subsequently, mice were sacrificed and their lungs collected for virus titration (Table 7). No signs of disease or major changes in body weight were observed in any of the mice after infection with 10^5^ EID_50_ or 10^7^ EID_50_ of any of the three viruses (data not shown). More importantly, replication of the QA23 and QA23CkA10 viruses in mice was clearly evident. All mice infected with the QA23 or QA23CkA10 viruses at doses of 10^5^ EID_50_ or 10^7^ EID_50_ had positive lung virus titers (Table 7). The average lung virus titers were 4.5 and 5.9 log_10_EID_50_ when mice were inoculated with 10^5^ EID_50_ or 10^7^ EID_50_, respectively, of the QA23 virus. Mice infected with 10^5^ EID_50_ or 10^7^ EID_50_ of the QA23CkA10 virus showed slightly lower titers with averages of 3.8 and 4.2 log_10_EID_50_, respectively. In contrast, no evidence of virus replication in the lungs was found in mice infected with the WT702 virus. Further evidence for the improved replication of the quail- and chicken-adapted viruses in mammalian cells was obtained by comparing the ability of these viruses to form plaques in MDCK cells versus CEK cells (Figs. 2A and B). The QA23 and QA23CkA10 viruses produced larger plaques in MDCK cells than the WT702 virus, whereas the three viruses were equally efficient at producing large similar sized plaques in CEK cells. In vitro growth kinetic studies showed that the QA23CkA10 virus replicates slightly better than the QA23 virus and better than WT702 in MDCK and CEK cells (Fig. 2B). Our studies provide direct evidence continued replication/circulation of a virus in this single avian species, the quail, could have a profound impact on the virus's ability to increase its host range. Alternatively, we can also speculate that adaptation in quail and chickens has selected for strains with improved replication efficiencies. However, the in vitro results do not necessarily explain why the QA23 virus produced higher virus titers in mice than the QA23CkA10 virus.

**Table 7:** Replication of H9N2 viruses mouse lung.

Inoculum dose (EID_50_/mouse)	Virus titers in lung homogenates (log_10_EID_50_/lung)^a^		
	WT702	QA23	QA23CkA10
1×10^3^	NT	<1.0	<1.0
1×10^5^	<1.0^b^	4.5±0.5	3.2±0.8
1×10^7^	<1.0	5.9±0.4	4.2±1.9

a Data are the average virus titer from the 4 mice lungs.

b , Indicates virus not detected in the 10-fold diluted samples inoculated into eggs.

NT, Not tested.

**Figure 3:**
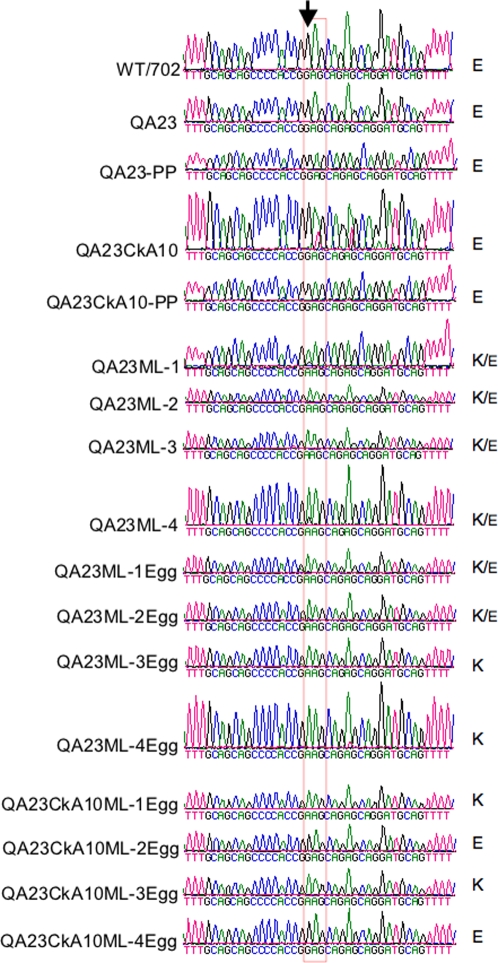
Comparison of nucleotide sequences of the PB2 gene near positions encoding for amino acid 627 between duck, quail- and chicken-adapted viruses before and after a single round of infection in mice. Alignment of electropherograms from PB2 sequences obtained from the WT702, QA23, and QA23CkA10 viruses before and after viruses were isolated from the lungs of mice. The arrowhead indicates the position of nucleotide change from G to A that generates the E627K mutation in PB2. E, K, or K/E, on the right of each electropherogram correspond to the predicted encoded amino acid for position 627 in the PB2 open reading frame. PP, sequence derived from plaque purified virus; ML, sequence derived from virus in mouse lung; ML-Egg, sequence derived from virus isolated from mouse lungs and amplified once in embryonated eggs.

A hallmark of influenza viruses that distinguishes those circulating in avian species from those established in humans is the presence of glutamic acid and lysine, respectively, at position 627 of the PB2 protein. Numerous studies have revealed that the E627K change in PB2 improves the virus's ability to replicate in mammalian cells. The K627 signature is also a marker of replication and high virulence in mice of some H5N1 strains. Since our quail- and chicken-adapted viruses that contained E627 in PB2 replicated efficiently in mice without prior adaptation, we were interested in determining whether such infection could result in the rapid selection of viral progeny carrying the E627K mutation. Sequencing analysis revealed that the WT702, QA23, and QA23CkA10 viruses encode for E627 in PB2 (Fig. 3). Alternatively, the QA23 and QA23CkA10 viruses were plaque purified and 2 independent clones were expanded in eggs. The viral clones were sequenced in the region spanning the nucleotides that encode for amino acid 627 in PB2 and confirmed the presence of E627 (Fig. 3). Each one of the clones from either QA23 or QA23CkA10 viruses revealed the presence of the GAG codon specific for glutamic acid (Fig. 3). Subsequently, the QA23 and QA23CkA10 viruses (not the plaque-purified versions) were selected to infect 4 mice. At 3 dpi, the lungs of each mouse were collected, viral RNA extracted and sequenced. Sequence results from the PB2 gene revealed that in each mouse infected with the QA23 virus there was the rapid selection of a viral progeny that carried a mutation at the first position of the codon encoding for amino acid position 627 in PB2. Thus, GAG was changed to AAG, which now encodes for lysine 627 instead of the glutamic acid present in the original virus inoculum (Fig. 3). A rapid E627K mutation was also observed in 2 out of the 4 mice analyzed that were previously infected with the QA23CkA10 virus. Detailed analysis of the sequencing profile suggests that, at least in some of the mouse lungs, a mixed population of viruses with E627 and K627 co-exist. These results suggest that adaptation of avian influenza viruses in land-based birds such as quail and chickens, not only can result in the selection of strains better adapted to replicate in mice but also in the selection of strains that can rapidly acquire mutations that favor replication in mammals.

**Figure 4:**
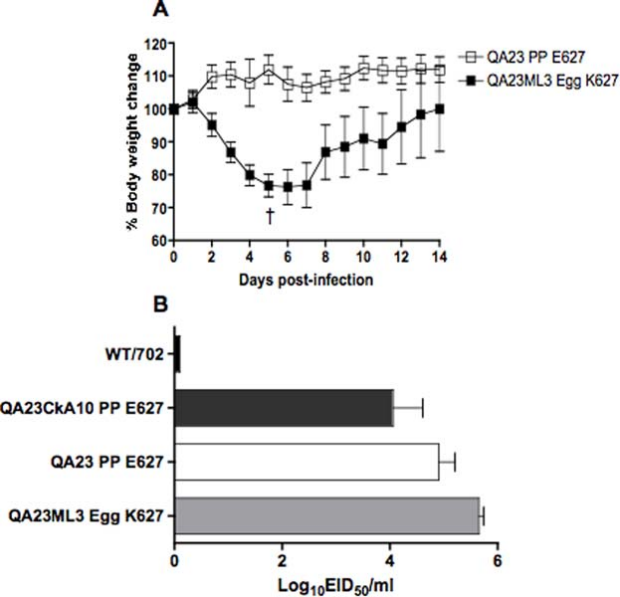
Body weight changes and lung virus titers in mice infected with H9N2 viruses. A) Groups of 4 mice were inoculated intranasally with 10^7^ EID_50_ of QA23ML-3 Egg (black squares) or QA23 PP (open squares) viruses. Body weight and clinical signs of disease were monitored for 14 dpi. Data presented correspond to average body weight changes in each group with corresponding standard deviations. †, one mouse infected with the QA23ML-3 Egg was humanely sacrificed due to severe disease signs. B) Virus titers in lung homogenates of mice (4/group) infected with 10^7^ EID_50_ of the WT702, QA23CkA10 PP, QA23 PP and QA23ML-3 Egg viruses as indicated. E627 and K627 correspond to the encoded amino acid position 627 in PB2.

The observation that a single round of infection in mice had such a profound effect on the selection of a virulence marker in PB2, prompted us to test whether acquisition of K627 would make these viruses more virulent for mice. Thus, one of the H9N2 viruses obtained from mouse lungs was further amplified in eggs (QA23ML-3Egg) and used to infect intranasally (10^7^ EID_50_) a group of 4 mice. Body weight changes and clinical signs of disease were monitored every day for 14 days (Fig. 4). Infections with the QA23ML3-Egg virus were compared to infections with plaque-purified (PP) versions of the QA23-PP and QA23CkA10-PP viruses (Fig. 4B). Mice infected with the QA23ML-3Egg virus showed clinical signs of disease and rapid body weight loss starting from 2 dpi, with approximately 28% body weight loss 4 days after infection, at which time one of the mice had to be humanely sacrificed. In contrast, mice infected with the QA23-PP virus (Fig 4A) and the QA23CkA10-PP virus (not shown) showed no body weight loss with no signs of disease. Alternatively, lung homogenates prepared at 4 dpi were used to determine virus titers. This analysis revealed that the K627-encoding virus produced higher and more consistent virus titers than any of the E627-containing viruses (Fig 4B). Interestingly, the QA23-PP and QA23CkA10-PP viruses produced slightly lower yields than their non-cloned counterparts, likely because they are more homogenous populations with no K627 subpopulations (Table 7 and Fig 4B). These studies are highly suggestive about the role of K627 in PB2 for virulence in mice as has been previously described. Sequence analysis of the QA23ML-3Egg revealed no other amino acid changes in the genome of the virus, except for the E627K mutation in PB2. Thus, we can conclude that this single amino acid mutation is solely responsible for the increase in virulence of the QA23ML-3Egg virus in mice. Our studies are in agreement with field observations and highlight the potential role of quail as an important contributor in the emergence of pandemic strains of influenza.

## Discussion

Surveillance studies in Asia have suggested that H9N2 viruses were circulating in healthy domestic waterfowl prior to 1990 [27]. The first evidence of H9N2 viruses in land-based poultry in Asia was during a disease outbreak in Japanese quail in Hong Kong in 1988 [20]. Since then, H9N2 viruses were regularly detected in land-based poultry in Asia and established several permanent lineages [28]–[30]. The continuous circulation of these viruses has had profound ecological and epidemiological implications since they have been involved in the emergence of H5N1 strains with expanded host range with the ability to infect humans and other mammals. More importantly, H9N2 viruses themselves have acquired features typical of human influenza viruses, such as the ability to bind efficiently α2,6 SA receptors and to replicate in human airway epithelial cultures in nonciliated cells [13], [31]. Furthermore, H9N2 viruses have infected humans causing mild respiratory disease while serology and epidemiological evidence suggested that pigs have been infected as well [8], [9], [12], [32]–[34]. Recent studies have also shown that some H9N2 viruses can replicate in mice without prior adaptation [14]–[16]. Adding to the pandemic potential of H9N2 viruses is the observation that strains closely related to those that crossed to humans and pigs continue to circulate in countries in Asia and the Middle East [14], [28]. These observations suggest that following the jump from waterfowl, H9N2 viruses circulated among land-based poultry for some time and progressively gained replication properties that made them more adapted to mammals. Our laboratory adaptation strategy in quail and chickens using a duck H9N2 virus and the subsequent realization that the virus replicated more efficiently in mice supports this hypothesis. The virus titers in the lungs of quail, chicken and mice infected with our laboratory adapted viruses are similar to those obtained with infection with field H9N2 isolates [14]–[16]. Previous studies have shown the potential of some avian influenza strains isolated from waterfowl to infect different mammalian species including pigs, ferrets, and cats; in these mammals very limited replication and lack of disease signs were observed [35], [36]. The prevailing notion is that avian influenza viruses must undergo molecular changes before they become better adapted to replicate in mammals [37], [38]. Our study is the first to demonstrate that such changes can be obtained through continued passages of the virus in a single avian species: the quail. Interestingly, quail have been implicated in the emergence of the first H9N2 virus that crossed to humans in 1999 and in contributing to the genetic make up of the first H5N1 that infected humans in 1997.

In one of our previous studies, we observed that a mallard H2N2 virus replicated poorly in quail and did not transmit to contact quail. Following adaptation in quail through serial lung passages (5 times), the adapted virus gained transmission properties in quail and replication/transmission properties in chickens [24]. In contrast, the duck H9N2 virus used in this study needed more passages in order to transmit to chickens. Does this mean that quail are not efficient in generating H9N2 viruses with the ability to replicate and transmit in mammals? It is impossible to predict the degree of fitness that a virus from the field may have for non-natural hosts. In this regard, our study highlights the complexities associated with adaptation of influenza viruses in different hosts and cautions against making dogmatic conclusions.

Several amino acid substitutions were identified scattered throughout the genome of the QA23 and QA23CkA10 viruses compared to the WT702 virus. It was also evident the loss of 21 amino acids spanning the stalk region of the NA protein in the QA23 and QA23CkA10 viruses. Once again, our studies show that quail is one of potentially many other land-based birds from which NA genes of various lengths could be produced. To our knowledge this is the longest amino acid deletion among the NA of H9N2 viruses to date based on the available public influenza sequence database. H9N2 viruses isolated from land based poultry often contain deletions in the stalk region of the NA; however these deletions are restricted to just a single or few amino acids [15], [39]. The avian H9N2 and H5N1 viruses that transmitted to humans in 1999 and 1997, respectively also contained deletions in their NA stalk regions [8], [40], [41]. The role of the NA stalk for replication and transmission of the H9N2 and H5N1 virus in different animal species is not yet clear; however, it has been suggested that a deletion in the NA could favor adaptation of the virus to land-based poultry [24], [40], [41]. This latter argument is consistent with our results and those obtained previously by us using a mallard H2N2 virus that was laboratory-adapted in quail resulting in a virus with a 27 amino acid deletion in the NA stalk [24]. More importantly, the deletion of 21 amino acids in the NA of the QA23 or QA23CkA10 virus was not an impediment for efficient replication in mice. It remains to be determined whether the deletion itself is sufficient to improve the virus's ability to replicate in poultry and mice.

Alignment of the deduced amino acid sequence of the adapted viruses with available published H9N2 sequences provided important clues regarding mutations in the HA protein: For example, most duck H9N2 viruses contain theronine at position 29 (40 in the H3 HA) in the HA1 region, whereas those from land-based poultry contain alanine. Likewise, most duck H9N2 viruses encode for glutamic acid at position 43 in the HA2 portion but most land-based poultry derived viruses encode for lysine [23]. In addition, our laboratory-adapted viruses contain one mutation in a region previously identified as potentially important in the adaptation of avian H9N2 viruses in land-based poultry (D133N in the HA2) [20]. Less can be speculated about the mutations elsewhere in the genome since they fall within regions with no specifically assigned functions, except for those identified in the NS1 protein. With only two exceptions, it must be noted that the changes observed throughout the genome are found in at least one other sequence available in the public domain, which suggest that our adaptation strategy mimics natural molecular events. It remains to be determined the biological significance of such changes for replication and transmission in quail and chickens and for replication in mice. It is worth mentioning the changes in NS1, which may be important for modulating the multiple NS1 activities in quail and chickens and may help the virus replicate in mice. Thus, it is tempting to speculate that quail, and perhaps other land-based birds, generate strains that have molecular changes more compatible with replication of influenza viruses in the respiratory tract of mammals. In this regard, it is important to mention that low pathogenic influenza infections in quail are mostly restricted to the respiratory tract [19]. The molecular mechanism(s) for adaptation of avian influenza viruses in mammalian hosts is still poorly understood. Several studies suggest that the presence of lysine at position 627 of PB2 favors the replication of influenza viruses in mammals and thus it is considered an important host range determinant [42]–[45]. Although K627 in PB2 is not the only marker for virulence of influenza viruses there is compelling evidence that the E627K change favors the replication and increases the virulence of some avian influenza viruses in mammals [46]. With regards to the 1997 Hong Kong H5N1 influenza viruses, the E627K mutation is attributed to provide growth advantages and increased pathogenicity in mice [47]. The H7N7 virus from the fatal human case in the Netherlands also possessed a lysine at this site, which is not shared by its closest avian counterpart [43]. There is also evidence for the rapid selection of H5N1 strains with K627 in PB2 after a single round of infection in mice, although the input virus may have had a mixture of viruses from which the K627 strains were selected out [48], [49]. Since we used plaque-purified viruses to infect mice in which the only discernible amino acid in position 627 was glutamic acid, we must conclude that the E627K mutation occurred during infection in mice and that it was rapidly selected out. This is consistent with the notion that the E627K change must provide some growth advantages for the virus in mice. Interestingly, some avian influenza isolates from domestic and wild birds, particularly H5N1 strains, carry lysine 627 in PB2, which implies that such change is tolerated among some bird species and/or that some bird species may act as a bridge in the generation of strains that can replicate more efficiently in mammals. In our particular case, quail provided an environment that improved the ability of a duck H9N2 virus to replicate in mice.

## Materials and Methods

### Viruses and cells

The A/Duck/Hong Kong/702/79 (H9N2) virus (WT702) was kindly provided by Dr. Robert G. Webster at St. Jude Children's Research Hospital, Memphis, TN. The quail-adapted (QA23) virus was isolated from the 23^rd^ quail lung passage. The chicken-adapted (QA23CkA10) virus was isolated from the 10^th^ chicken lung passage of the QA23 virus. Stocks of QA23 and QA23CkA10 viruses were prepared by the inoculation of lung homogenates into the allantoic cavity of 10-day-old embryonated chicken eggs. Subsequently, the QA23 and QA23CkA10 viruses were plaque-purified using confluent Madin-Darby canine kidney (MDCK) cells. MDCK cells were regularly maintained in Modified Eagle's medium (MEM) (Sigma-Aldrich, St. Louis, MO) containing 5% fetal bovine serum (FBS) (Sigma-Aldrich). Four days following the plaque assay, a single plaque from each virus was isolated and used for a second round of plaque purification in MDCK cells. Following 2-plaque purifications, 2 plaques from each virus were grown in embryonated eggs to produce a working stock of virus. Alternatively, viruses were grown in chicken embryo kidney (CEK) cells where specified. CEK cells were prepared from 17-days-old chicken embryo according to the method described previously [13].

### In vitro growth kinetics of wild type and adapted H9N2 viruses

Growth kinetics of WT702 and plaque purified QA23 and QA23CkA10 viruses were monitored in MDCK cells and CEK cells. Confluent monolayers of MDCK or CEK cells were infected with the viruses at a multiplicity of infection (m.o.i) of 0.001. Following 1 h adsorption at 37°C, cells were washed three times with HBSS (Sigma-Aldrich) containing antibiotic/antimycotic. Cells were then maintained in Opti-MEM (Invitrogen, Long Island, NY) or Medium 199 (Sigma-Aldrich) supplemented with TPCK-trypsin (Worthington Biochemical Co., Lakewood, NJ) at a concentration of 1 µg/ml. Supernatants were collected every 12 h for 72 hpi and stored at −80°C following clarification by centrifugation. Virus titers in the supernatant were measured by TCID_50_ in CEK cells according to the method described previously [13]. Plaque assays in MDCK and CEK cells were performed as previously described [50].

### Animals and experimental infections

4- to 6-weeks old Japanese quail (*Coturnix coturnix*, University of Maryland, College Park, Central Research Facility) and 3 to 4-weeks old specific-pathogen free White Leghorn chickens (Charles River Laboratories, Wilmington, MA) were used throughout the study. Groups of three birds were inoculated intraocularly, intranasally, intratracheally and orally with different doses of virus diluted in 0.6 ml (quail) or 1.0 ml (chicken) in PBS. Tracheal and cloacal swabs were collected at 1, 3, 5, 7, 9, 11, and 13 dpi and were stored in 1 ml freezing medium at −70°C (50% glycerol in PBS containing 1% antibiotics). Virus titers in the swabs were determined by EID_50_ as previously described. Birds were observed and scored daily for clinical signs of infection and general well being. They were evaluated on appetite, activity, fecal output and signs of distress including cyanosis of the tongue or legs, ruffled feathers and respiratory distress. Experiments were carried out under BSL-2 conditions with investigators wearing appropriate protective equipment and compliant with all Institutional Animal Care and Use Committee (IACUC) of University of Maryland approved protocols and under the Animal Welfare (AWA) regulations.

### Serial lung passages of H9N2 viruses in quail and chickens

For quail lung passages, quail were inoculated with the WT702 virus (5×10^6^ EID_50_) by the ocular, nasal, oral and tracheal routes. Three quail were used for each passage. At 3 dpi, quail were sacrificed and lung homogenates were prepared using a mortar and sterile sea sand. The homogenates were resuspended in 3 ml sterile brain heart infusion (BHI) medium containing antibiotics as described [51]. Then the homogenate was clarified by centrifugation at 2,000 rpm for 15 min at 4°C and stored at −80°C until used. For serial lung passages, lung homogenates from 3 individual quail were pooled and quail were inoculated with 600 µl by the nasal, oral and tracheal routes. The WT702 virus was serially passaged 23 times in quail (QA23). Chickens were initially infected with the QA23 at a concentration of 1×10^8^ EID_50_. At 3 dpi, lung homogenates were prepared as described above. Pooled lung homogenates (1 ml) prepared from 3 separate chickens were used to inoculate the next group of chickens by nasal, oral, tracheal and cloacal routes. After 10 sequential lung passages in chickens (QA23CkA10), a stock virus was prepared in 10-days-old embryonated chickens eggs.

### Replication and transmission of H9N2 influenza viruses in quail and chickens

For replication and transmission studies, groups of 3 quail or chickens were inoculated with 5×10^6^ or 1×10^8^ EID_50_ in 0.6 ml or 1 ml PBS, respectively. The birds were inoculated by the ocular, nasal, oral and tracheal routes. At 1 dpi, 3 naïve birds were placed together with the infected birds. Tracheal and cloacal swabs were collected at 1, 3, 5, 7, 9, 11 and 13 dpi. To investigate replication of WT702, QA23 and QA23CkA10 viruses in the tissues of quail (lung) and chickens (lung and intestine) a group of 3 birds were sacrificed at 3 dpi and tissue homogenates were prepared in BHI medium and stored at −80°C. Virus shedding through trachea and cloaca were monitored by the inoculation of swabs into 10-days-old embryonated chicken eggs. The virus titers in lung homogenates were measured also by the inoculation into 10-days-old embryonated chicken eggs.

### Replication of H9N2 virues in mice

5-week-old female BALB/c mice, 4 per group, were infected intranasally with different doses of the WT702, QA23, QA23-PP, QA23CkA10, QA23CkA10-PP, and QA23ML-3 Egg viruses in 50 µL PBS under light anesthesia using isoflurane (Abbot Laboratories, North Chicago, IL). Body-weight and signs of disease were monitored everyday until they were sacrificed (4 or 14 dpi as indicated). Lung homogenates and virus titers were determined as described above.

### Isolation of RNA, reverse transcription-PCR amplification, and sequencing

Total RNA was extracted from stock virus in allantoic fluid or from the mouse lung homogenate using the Rneasy kit (Qiagen, Valencia, CA, USA) according to manufacturer's instructions. Reverse transcription was carried out with the uni-12 primer (5′-AGCAAAAGCAAAGG-3′) and AMV reverse transcriptase (Promega, Madison, WI, USA). PCR amplification was performed using the universal primers described by Hoffman et al. [52] as well as specific primers. The PCR products were sequenced using BigDye-Terminator protocol V3.1 (Applied Biosystems, Foster City, CA, USA). Full genome sequences were determined, except for regions recognized by the 5′ and 3′ end universal primers. Sequences have been deposited in GenBank with accession numbers CY031256 through CY031287

## References

[1] Homme PJ, Easterday BC (1970). Avian influenza virus infections. I. Characteristics of influenza A-turkey-Wisconsin-1966 virus.. Avian Dis.

[2] Aamir UB, Wernery U, Ilyushina N, Webster RG (2007). Characterization of avian H9N2 influenza viruses from United Arab Emirates 2000 to 2003.. Virology.

[3] Alexander DJ (2007). An overview of the epidemiology of avian influenza.. Vaccine.

[4] Guo Y, Li J, Cheng X (1999). [Discovery of men infected by avian influenza A (H9N2) virus].. Zhonghua Shi Yan He Lin Chuang Bing Du Xue Za Zhi.

[5] Kim JA, Cho SH, Kim HS, Seo SH (2006). H9N2 influenza viruses isolated from poultry in Korean live bird markets continuously evolve and cause the severe clinical signs in layers.. Vet Microbiol.

[6] Naeem K, Ullah A, Manvell RJ, Alexander DJ (1999). Avian influenza A subtype H9N2 in poultry in Pakistan.. Vet Rec.

[7] Nili H, Asasi K (2003). Avian influenza (H9N2) outbreak in Iran.. Avian Dis.

[8] Lin YP, Shaw M, Gregory V, Cameron K, Lim W (2000). Avian-to-human transmission of H9N2 subtype influenza A viruses: relationship between H9N2 and H5N1 human isolates.. Proc Natl Acad Sci U S A.

[9] Peiris JS, Guan Y, Markwell D, Ghose P, Webster RG (2001). Cocirculation of avian H9N2 and contemporary “human” H3N2 influenza A viruses in pigs in southeastern China: potential for genetic reassortment?. J Virol.

[10] Butt KM, Smith GJ, Chen H, Zhang LJ, Leung YH (2005). Human infection with an avian H9N2 influenza A virus in Hong Kong in 2003.. J Clin Microbiol.

[11] Peiris M, Yam WC, Chan KH, Ghose P, Shortridge KF (1999). Influenza A H9N2: aspects of laboratory diagnosis.. J Clin Microbiol.

[12] Xu C, Fan W, Wei R, Zhao H (2004). Isolation and identification of swine influenza recombinant A/Swine/Shandong/1/2003(H9N2) virus.. Microbes Infect.

[13] Wan H, Perez DR (2007). Amino Acid 226 in the Hemagglutinin of H9N2 Influenza Viruses Determines Cell Tropism and Replication in Human Airway Epithelial Cells.. J Virol.

[14] Choi YK, Ozaki H, Webby RJ, Webster RG, Peiris JS (2004). Continuing evolution of H9N2 influenza viruses in Southeastern China.. J Virol.

[15] Guo YJ, Krauss S, Senne DA, Mo IP, Lo KS (2000). Characterization of the pathogenicity of members of the newly established H9N2 influenza virus lineages in Asia.. Virology.

[16] Li C, Yu K, Tian G, Yu D, Liu L (2005). Evolution of H9N2 influenza viruses from domestic poultry in Mainland China.. Virology.

[17] Wan H, Sorrell EM, Song H, Hossain MJ, Ramirez-Nieto G (2008). Replication and transmission of H9N2 influenza viruses in ferrets: evaluation of pandemic potential.. PLoS ONE.

[18] Liu M, He S, Walker D, Zhou N, Perez DR (2003). The influenza virus gene pool in a poultry market in South central china.. Virology.

[19] Makarova NV, Ozaki H, Kida H, Webster RG, Perez DR (2003). Replication and transmission of influenza viruses in Japanese quail.. Virology.

[20] Perez DR, Lim W, Seiler JP, Yi G, Peiris M (2003). Role of quail in the interspecies transmission of H9 influenza A viruses: molecular changes on HA that correspond to adaptation from ducks to chickens.. J Virol.

[21] Wan H, Perez DR (2006). Quail carry sialic acid receptors compatible with binding of avian and human influenza viruses.. Virology.

[22] Perez DR, Webby RJ, Hoffmann E, Webster RG (2003). Land-based birds as potential disseminators of avian mammalian reassortant influenza A viruses.. Avian Dis.

[23] Guan Y, Shortridge KF, Krauss S, Webster RG (1999). Molecular characterization of H9N2 influenza viruses: were they the donors of the “internal” genes of H5N1 viruses in Hong Kong?. Proc Natl Acad Sci U S A.

[24] Sorrell EM, Perez DR (2007). Adaptation of influenza A/Mallard/Potsdam/178-4/83 H2N2 virus in Japanese quail leads to infection and transmission in chickens.. Avian Dis.

[25] Kochs G, Garcia-Sastre A, Martinez-Sobrido L (2007). Multiple anti-interferon actions of the influenza A virus NS1 protein.. J Virol.

[26] Melen K, Kinnunen L, Fagerlund R, Ikonen N, Twu KY (2007). Nuclear and nucleolar targeting of influenza A virus NS1 protein: striking differences between different virus subtypes.. J Virol.

[27] Shortridge KF (1992). Pandemic influenza: a zoonosis?. Semin Respir Infect.

[28] Cameron KR, Gregory V, Banks J, Brown IH, Alexander DJ (2000). H9N2 subtype influenza A viruses in poultry in pakistan are closely related to the H9N2 viruses responsible for human infection in Hong Kong.. Virology.

[29] Lee CW, Song CS, Lee YJ, Mo IP, Garcia M (2000). Sequence analysis of the hemagglutinin gene of H9N2 Korean avian influenza viruses and assessment of the pathogenic potential of isolate MS96.. Avian Dis.

[30] Guan Y, Shortridge KF, Krauss S, Chin PS, Dyrting KC (2000). H9N2 influenza viruses possessing H5N1-like internal genomes continue to circulate in poultry in southeastern China.. J Virol.

[31] Matrosovich MN, Krauss S, Webster RG (2001). H9N2 influenza A viruses from poultry in Asia have human virus-like receptor specificity.. Virology.

[32] Ninomiya A, Takada A, Okazaki K, Shortridge KF, Kida H (2002). Seroepidemiological evidence of avian H4, H5, and H9 influenza A virus transmission to pigs in southeastern China.. Vet Microbiol.

[33] Peiris M, Yuen KY, Leung CW, Chan KH, Ip PL (1999). Human infection with influenza H9N2.. Lancet.

[34] Saito T, Lim W, Suzuki T, Suzuki Y, Kida H (2001). Characterization of a human H9N2 influenza virus isolated in Hong Kong.. Vaccine.

[35] Hinshaw VS, Webster RG, Easterday BC, Bean WJ (1981). Replication of avian influenza A viruses in mammals.. Infect Immun.

[36] Kida H, Ito T, Yasuda J, Shimizu Y, Itakura C (1994). Potential for transmission of avian influenza viruses to pigs.. J Gen Virol.

[37] Murphy BR, Hinshaw VS, Sly DL, London WT, Hosier NT (1982). Virulence of avian influenza A viruses for squirrel monkeys.. Infect Immun.

[38] Beare AS, Webster RG (1991). Replication of avian influenza viruses in humans.. Arch Virol.

[39] Xu KM, Li KS, Smith GJ, Li JW, Tai H (2007). Evolution and molecular epidemiology of H9N2 influenza A viruses from quail in southern China, 2000 to 2005.. J Virol.

[40] Matrosovich M, Zhou N, Kawaoka Y, Webster R (1999). The surface glycoproteins of H5 influenza viruses isolated from humans, chickens, and wild aquatic birds have distinguishable properties.. J Virol.

[41] Li KS, Guan Y, Wang J, Smith GJ, Xu KM (2004). Genesis of a highly pathogenic and potentially pandemic H5N1 influenza virus in eastern Asia.. Nature.

[42] Subbarao EK, London W, Murphy BR (1993). A single amino acid in the PB2 gene of influenza A virus is a determinant of host range.. J Virol.

[43] Fouchier RA, Schneeberger PM, Rozendaal FW, Broekman JM, Kemink SA (2004). Avian influenza A virus (H7N7) associated with human conjunctivitis and a fatal case of acute respiratory distress syndrome.. Proc Natl Acad Sci U S A.

[44] Puthavathana P, Auewarakul P, Charoenying PC, Sangsiriwut K, Pooruk P (2005). Molecular characterization of the complete genome of human influenza H5N1 virus isolates from Thailand.. J Gen Virol.

[45] Naffakh N, Massin P, Escriou N, Crescenzo-Chaigne B, van der Werf S (2000). Genetic analysis of the compatibility between polymerase proteins from human and avian strains of influenza A viruses.. J Gen Virol.

[46] Li Z, Chen H, Jiao P, Deng G, Tian G (2005). Molecular basis of replication of duck H5N1 influenza viruses in a mammalian mouse model.. J Virol.

[47] Hatta M, Gao P, Halfmann P, Kawaoka Y (2001). Molecular basis for high virulence of Hong Kong H5N1 influenza A viruses.. Science.

[48] Mase M, Eto M, Tanimura N, Imai K, Tsukamoto K (2005). Isolation of a genotypically unique H5N1 influenza virus from duck meat imported into Japan from China.. Virology.

[49] Lipatov AS, Krauss S, Guan Y, Peiris M, Rehg JE (2003). Neurovirulence in mice of H5N1 influenza virus genotypes isolated from Hong Kong poultry in 2001.. J Virol.

[50] Song H, Nieto GR, Perez DR (2007). A new generation of modified live-attenuated avian influenza viruses using a two-strategy combination as potential vaccine candidates.. J Virol.

[51] Spackman E, Senne DA, Bulaga LL, Myers TJ, Perdue ML (2003). Development of real-time RT-PCR for the detection of avian influenza virus.. Avian Dis.

[52] Hoffmann E, Krauss S, Perez D, Webby R, Webster RG (2002). Eight-plasmid system for rapid generation of influenza virus vaccines.. Vaccine.

